# Functional Redundancy in bird community decreases with riparian forest width reduction

**DOI:** 10.1002/ece3.4448

**Published:** 2018-10-11

**Authors:** Lucas A. Maure, Rodolpho C. Rodrigues, Ângelo V. Alcântara, Bruno F. C. B. Adorno, Douglas L. Santos, Eduardo L. Abreu, Rafael M. Tanaka, Rute M. Gonçalves, Erica Hasui

**Affiliations:** ^1^ Instituto de Ciências da Natureza Universidade Federal de Alfenas Alfenas Minas Gerais Brazil; ^2^ Departamento de Ecologia Instituto de Biociências Universidade de São Paulo São Paulo São Paulo Brazil

**Keywords:** bird communities, disturbance, fragmentation, functional diversity, functional evenness, functional redundancy

## Abstract

Riparian ecosystems are suffering anthropogenic threats that reduce biodiversity and undermine ecosystem services. However, there is a great deal of uncertainty about the way species composition of assemblages is related to ecosystem function, especially in a landscape fragmentation context.
Here, we assess the impact of habitat loss and disturbance on Functional Diversity (FD) components Functional Redundancy (FRed), Functional Evenness (FEve), and Functional Richness (FRic) of riparian forest bird assemblages to evaluate (a) how FD components respond to riparian forest width reduction and vegetation disturbance; (b) the existence of thresholds within these relationships; (c) which of the main birds diet guild (frugivores, insectivores, and omnivores) respond to such thresholds. We predict that FD components will be affected negatively and nonlinearly by riparian changes. However, guilds could have different responses due to differences of species sensitivity to fragmentation and disturbance. We expect to find thresholds in FD responses, because fragmentation and disturbance drive loss of specific FD components.
Our results show that FRed and FEve were linearly affected by width and disturbance of riparian habitats, respectively. FRed was significantly lower in riparian forests assemblages below 400 m wide, and FEve was significantly higher above 60% disturbance. These responses of FD were also followed to the decline in insectivores and frugivores richness in riparian forests most affected by these changes.
Consequently, our study suggests communities do not tolerate reduction in riparian forest width or disturbance intensification without negative impact on FD, and this becomes more critical for riparian area <400‐m wide or with more than 60% disturbance. This minimum riparian width required to maintain FRed is greater than the minimum width required for riparian forests by Brazilian law. Thus, it is important to consider mechanisms to expand riparian habitats and reduce the disturbance intensity in riparian forests so that riparian bird community FD may be effectively conserved.

## 1 INTRODUCTION

Riparian forests play a key role in ecology and biogeography of South America vertebrates (Cardoso Da Silva & Bates, 2002; Nogueira, Ribeiro, Costa, & Colli, 2011; Redford & Fonseca, 1986; Tubelis, Cowling, & Donnelly, 2004). They are also important for the maintenance of biodiversity in fragmented landscapes, due to their function as both primary habitat and migratory corridors for many biological groups (Bueno, Bruno, Pimentel, Sanaiotti, & Magnusson, 2012; Dobkin, Rich, & Pyle, 1998; Lees & Peres, 2008; Monadjem, 2005; Monadjem & Reside, 2008). Additionally, riparian habitats provide a variety of ecosystem services, including the protection of water sources for drinking and irrigation, flood regulation, soil regeneration, and the provision of habitat for pollinators, seed dispersers and biological controllers (Holmes, Bergstrom, Huszar, Kask, & Orr, 2004; Power, 2010).

However, both agricultural and urban expansions have strongly impacted riparian forests causing declines in biodiversity and undermining ecosystem services. For instance, fragmentation and reduction in the width of riparian forests can alter community structure, reducing frugivorous and insectivorous bird abundance, and increasing that of omnivores and granivores (Banks‐leite et al., 2012; Bueno et al., 2012; Gray, Baldauf, Mayhew, & Hill, 2007; Metzger & Décamps, 1997; de Oliveira Ramos & dos Anjos, 2014). Such community changes may have detrimental impacts on ecosystem functioning and properties and ultimately impact seed dispersion and pest control ecosystem services functionality (Balvanera et al., 2006, 2014; Cardinale et al., 2012; Larsen, Williams, & Kremen, 2005; Power, 2010; Sekercioglu, 2006). In consequence, the importance of these negative impacts has increased interest in describing the relationships between ecosystem functioning and biological diversity and in understanding how to maintain the stability of ecosystem properties under conditions of anthropogenic threat (Maestre et al., 2012; Tilman, Isbell, & Cowles, 2014).

Several studies have proposed functional forms for biodiversity‐ecosystem relationships (Naeem, Kawabata, & Loreau, 1998). Some of these have found a positive linear relationship between species richness and ecosystem function, suggesting that every species plays a unique role in the functioning of the ecosystem (Schwartz, Brigham, & Hoeksema, 2000), and all species are required to maintain healthy levels of ecosystem function (Johnson, Vogt, Clark, Schmitz, & Vogt, 1996). However, most studies found an asymptotic relationship among those variables and suggest that above a critical number of species, the addition of new species adds more redundancy than novel capacities to specific functions (Schwartz et al., 2000; Vitousek & Hooper, 1994). This asymptotic relationship is potentially explained by the Ecosystem Redundancy (Walker, 1992) and Insurance hypotheses (Yachi & Loreau, 1999). The central idea behind these is that there is a minimum number of species required for effective ecosystem functioning, to provide a buffer against environmental disturbance and/or resilience for reorganization after disturbance events (Loreau et al., 2002; Mori, Furukawa, & Sasaki, 2013). On the other hand, when species richness drops below this threshold, ecosystem functioning may be disrupted and abrupt local species extinctions are more likely to occur (Alados et al., 2004; Zavaleta, 2004).

Thus, there is a great deal of uncertainty about the way biodiversity in terms of species richness and complexity is related to ecosystem function. Nevertheless, research in the last two decades has successfully studied this relationship in disturbed habitats using a functional trait approach (Díaz & Cabido, 2001; Díaz et al., 2007, 2013; Lavorel & Garnier, 2002). Such studies use species traits to calculate Functional Diversity (FD) components and related them to species and community responses to disturbance (response traits), as well as the disturbance effects on ecosystem processes (effect traits) (Mouillot, Graham, Villéger, Mason, & Bellwood, 2013; Suding et al., 2008). Functional Diversity metrics are based on the distance of trait values distributed in a functional space and have been considered a viable surrogate for ecosystem functioning, as it expresses the species influence on ecological processes, and how resources are utilized by different organisms (Cadotte, Carscadden, & Mirotchnick, 2011; Díaz & Cabido, 2001; Hooper et al., 2005; Mason, Mouillot, Lee, & Wilson, 2005; Mouillot et al., 2013; Villéger, Mason, & Mouillot, 2008). Thus, FD components, such as Functional Redundancy (FRed), Functional Evenness (FEve), and Functional Richness (FRic), bring a new perspective to community responses to land use intensification and habitat disturbance (Flynn et al., 2009; Mouillot et al., 2013). Although there is little evidence that relates FD to ecosystem function in a landscape fragmentation context (Hatfield, Harrison, & Banks‐leite, 2018), it is generally expected that Functional Diversity components will decline with intensification of disturbance and fragmentation processes (Hatfield et al., 2018; Mouillot et al., 2013).

In this study, we assessed the impact of habitat loss and disturbance on Functional Diversity of riparian forest bird communities in southeastern Brazil. Specifically, we estimated three components of FD, namely FRed, FEve, and FRic, to evaluate: (a) how these FD components respond to riparian forest changes (i.e., habitat and vegetation disturbance and changes in riparian forest width); (b) the existence of critical thresholds (i.e., sudden change in FD metrics along disturbance gradients) in these relationships, and; (c) which of the main functional groups of birds responds to these thresholds. We predict that FD components will be affected negatively and nonlinearly by riparian changes. However, functional groups could have different responses due to variance in species sensitivity to fragmentation and disturbance (Gray et al., 2007; de Oliveira Ramos & dos Anjos, 2014). We expect to find thresholds in Functional Diversity responses, because fragmentation and disturbance drive loss in some Functional Diversity components (Ding, Feeley, Wang, Pakeman, & Ding, 2013; Magnago et al., 2014; Mouillot et al., 2013). As species traits confer on species differences in their ability to tolerate habitat changes, we also expected alterations in community composition with respect to functional groups to occur along gradients of riparian forest width and disturbance (De Coster, Banks‐Leite, & Metzger, 2015; Mouillot et al., 2013). Hence, if our expectations are correct, we could use these thresholds to define the minimum width and structural integrity required for riparian forests in the human‐modified landscapes to avoid drastic changes in Functional Diversity of some or all functional groups.

## 2 MATERIALS AND METHODS

### 2.1 Study area

**Figure 1:**
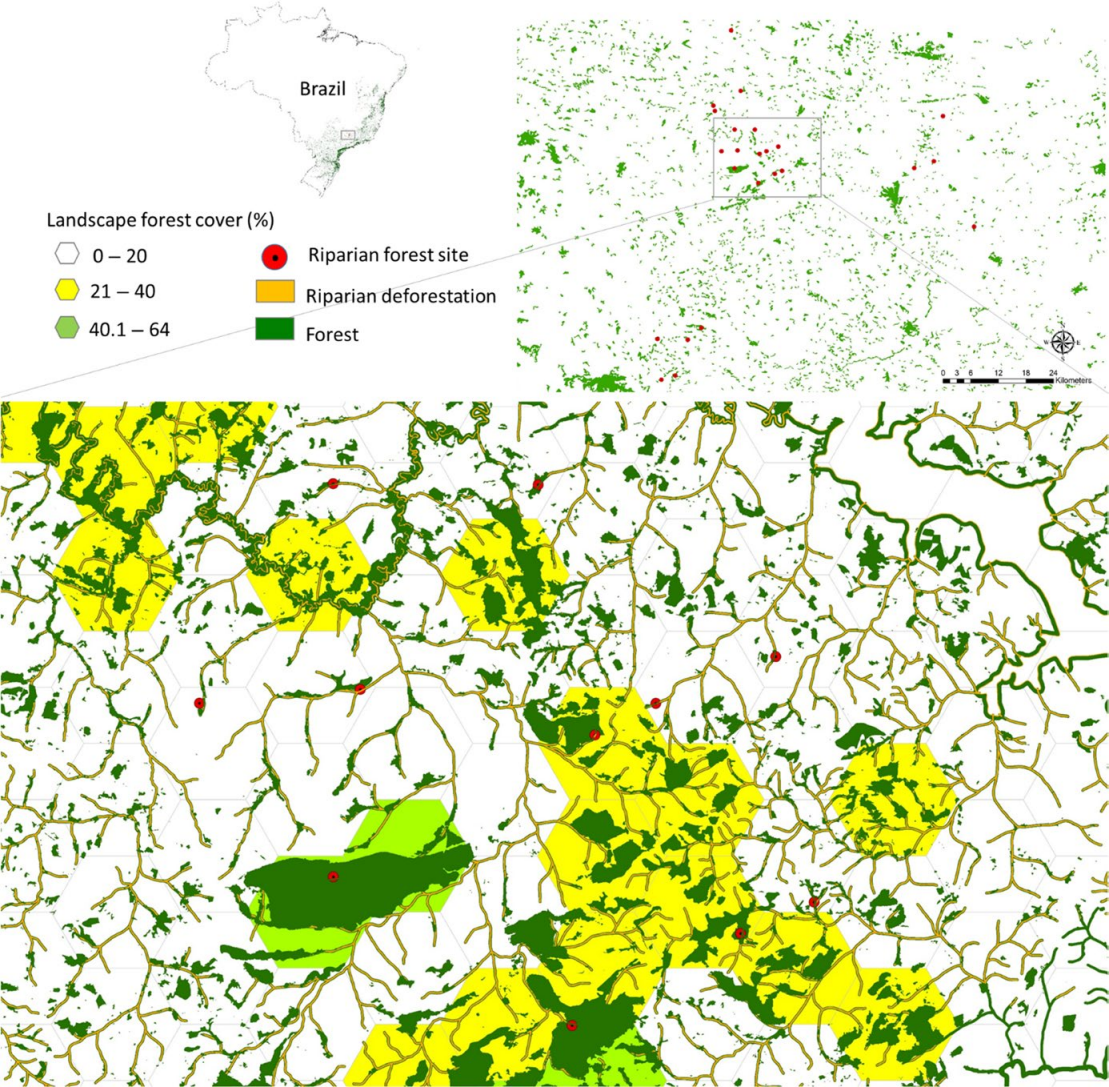
Study site map in semideciduous seasonal Atlantic Rainforest in southeastern Minas Gerais State, southeastern Brazil. The study site contained 24 riparian forests (red circles), which varied from 30 to 1,420 m in width (considering both stream banks)

We performed our study in 24 riparian forests located in southern Minas Gerais state, southeastern Brazil (Figure 1, Supporting Information Table S1). Regional climate is classified as Cwa (humid subtropical climate) by Köppen‐Geiger system, with a tropical rainfall pattern in summer and dry winters. Average annual temperature is 20.2°C, and annual precipitation being around 1,516 mm (available in https://en.climate-data.org/info/sources/). The relief is formed by gentle hills that are primarily structured formed by old orogens and the altitudes are often >800 m a. s. l.

The studied forest remnants lie in a transitional region between the Atlantic Forest and Cerrado, two of the most biologically rich and highly threatened biomes in Brazil (Myers, Mittermeier, Mittermeier, da Fonseca, & Kent, 2000). The original forest type in this region is described as semideciduous seasonal Atlantic Rainforest (IBGE 2012), but in much of the region, this has been drastically reduced to small and sparse forest fragments. In the study area, 99% of remaining forest patches are smaller than 20 ha, and 78% of the landscape in which these patches occur have forest cover below 20%. Even with specific protection laws for riparian forests (New Forest Code (Law 12,651/2012: available at: http://saema.com.br/files/Novo%20Codigo%20Florestal.pdf), almost 75% of these habitats are deforested and used for pasture, coffee, and sugarcane plantations or other agricultural uses (E. Hasui, personal communication).

### 2.2 Riparian forest selection

We selected the riparian forest sites by combining information from land cover and drainage network maps. The land cover map was built by unsupervised classification (oriented object method) of six RapidEye satellite images (5‐m resolution, total area = 3,586 km²) from 2010 using ENVI‐EX 4.8 software. To assess the land cover map accuracy, we performed a visual inspection using Google Earth satellite images and validated this information with field visits. The drainage network map was digitalized from topographic maps (1:50.000 scales) produced by IBGE (available at http://www.ibge.gov.br/home/geociencias/download/arquivos/index1.shtm). We identified riparian forests by then using this map to produce images containing buffers 15‐m wide beside each stream and 200 m around the Furnas water reservoir.

Next, we divided the region in 102 landscape hexagons (each landscape area = 500 ha) and measured patch and landscape metrics (patch size, shape, riparian forest width, and percentage of forest cover) for each fragment lying within these landscapes. We used these metrics to select 24 riparian forests that varied widely in riparian forest width and local and landscape contexts. Those selected sites ranged from 30 to 1,420 m in width considering both sides of the stream, from 0.88 to 496.18 ha in patch size from 3% to 67% in forest cover, presented a similar forest shape (we excluded complex forms or riparian corridors connected to other forest fragments), and were separated by at least 2 km from each other (Supporting Information Table S1). We did not use forest cover percentage and patch size in subsequent regression analyses due to the high correlations between these metrics and riparian forest width (Pearson correlation, *r* > 0.8).

### 2.3 Predictor variables

In addition to width, we used four disturbance indices to evaluate the disturbance levels at our riparian forest study sites:

*Disturbance index (%)*: We created a disturbance index that expressed the relative local disturbance intensity at each riparian forest. Within each bird sampling plot (30 m radius), we measured the number of vertical forest layers, maximum vegetation height, percentage of canopy cover and the frequencies of epiphytes, vines (i.e., herbaceous or sub‐woody climbing plants, which commonly grow in disturbed places or forest edges), and invasive plant species (i.e., *Brachiaria* spp.) (Imaflora 2008). We counted the number of vertical stratification layers (i.e., emergent, canopy, understory, shrub, herbaceous ground cover), using the maximum height of the different trees, ontogenetic stages, and plant life form. Usually, preserved forests had greater proportions of tall trees (height 20–25 m), more vertical strata (>2 strata), a more closed canopy, presence of young individuals of tree species in the understory forest, and higher epiphyte frequency (i.e., Cactaceae, Bromeliaceae, and Orchidaceae). In contrast, disturbed forests were characterized by more open canopy, fewer strata (1–2 strata), higher densities of short trees (1–15 m), higher frequencies of vines, and presence of invasive species. We standardized these data to reduce the variance between them and used principal components analysis (PCA) to summarize information for these vegetation variables into one orthogonal variable (first axis) (Zar, 1996). The first PCA axis was positively affected by the number of vertical strata, canopy cover, total height and was negatively affected by the frequency of invasive plant species (Supporting Information Table S3). It accounted for 45.4% of the total variance in vegetation measurements (Eigenvalue = 2.73) and carried information about vegetation structure and disturbance conditions of the sample points in riparian forests. Therefore, higher values can be interpreted as well‐protected old‐growth forest and lower values as young‐regenerating forests or forest growing under regularly disturbed conditions. Prior to further using this variable, we transformed it with Min‐Max‐Scaling means (Mf‐ min Mf)*100/(max Mf – min Mf) to standardize the range of values between 0 and 100. After this, we subtracted 100 from each value. Thus, disturbance index = 100% is the most disturbed condition and disturbance index = 0% is the least impacted condition.
*NDVI mean*: We used dry season (cloud‐free) Landsat TM 5 2011 images to calculate the normalized difference vegetation index (NDVI) for each riparian forest cell (30 m resolution) using the formula: NDVI = (NIR − red)/(red + NIR) (Marabel & Alvarez‐Taboada, 2013). Then, we used zonal statistical analysis to calculate NDVI values for each riparian study forest. NDVI is sensitive to photosynthetically active biomass and is positively correlated with plant productivity (Pettorelli et al., 2005). NDVI values range from −1.0 (indicate nonvegetated surface features such as water, barren rock) to 1.0 (maximum green vegetation). Thus, the maximum values are indicative of riparian forests possessing fully recovered above‐ground biomass levels (old‐growth forests), while lower values indicate low values for, or absence of, photosynthetic activity due to the existence of canopy gaps, young regrowth, or extensive edge effects.
*NDVI range*: Within a given riparian forest, spatial and biological disturbance can create a mosaic of patches at different successional stages, implying spatial variation in the timing or intensity of disturbance, and consequently varying capacities for ecosystem services delivery (Ferraz et al., 2014). To represent this spatial pattern, we used NDVI range values for each riparian forest for 2011. This index expresses the spatial heterogeneity of riparian forest in terms of plant productivity (i.e., above‐ground biomass). Thus, higher values indicate that riparian forest is composed by a mosaic of patches at different successional stages, whereas narrow range of NDVI point to a greater homogeneity in photosynthetic activity within areas.
*NDVI coefficient* β: We created this index to express the “historical dynamic” or “the resilience” of the riparian forest as the slope of the curve (coefficient β) of NDVI per unit time (i.e., per year). Here, high positive values mean that riparian forests possess periods of higher growth rates, while low values indicate periods of lower growth rates or higher stability in primary productivity (Viedma, Meliá, Segarra, & García‐Haro, 1997). Negative values represent forest degradation or loss over the time. To calculate this index, we used 10 Landsat Tm 5 dry season images from 1985 to 2011. First, we used zonal statistical analysis to calculate mean normalized difference vegetation index (NDVI) values for each riparian forest in each image. Then, we applied linear regression between the mean NDVI values by year and estimated the slope of the curve of this relationship for each riparian forest site (Supporting Information Figure S1).

### 2.4 Bird sampling

We used point counts to sample birds (Develey, 2003). We selected three stream‐side point count locations in each riparian forest, with each point separated by at least 200 m. Each sampling location was visited once between 2013 and 2017, and we defined as the sampling area a zone of 30‐m radius around each point. Sampling observations occurred always in the three first hours after sunrise and consisted in recording all individuals seen and/or heard inside the 30‐m buffer during 10 min of observation at each point. We excluded Trochilidae (hummingbirds) from our samples because of the difficulty of identifying them using point counts. We calculated the relative abundance of each species by adding the number of all individuals observed (avoiding double counting of the same individuals) in the three point locations of each riparian forest.

For each sampled species, we compiled information of morphological traits and foraging attributes (Table 1) that are related to bird species function in the ecosystem (Flynn et al., 2009). We chose traits reflecting habitat preferences and resource use requirements, including body size, diet, foraging habitat, and location (Wilman et al., 2014; Birdlife database *available at* http://www.birdlife.org/datazone/species/search). We define a functional group as a set of species that respond similarly to a particular habitat condition.

**Table 1:** List and description of traits used to calculate predictive variables (Functional Redundancy, Functional Evenness, and Functional Richness) and define functional group richness

Trait	Scale	Description	Ecological relevance to ecosystem process	Source
Diet (seven items)	Continuous	Based on the percentage contribution of each food item to the total dietary records for the species: seeds, fruits, nectar, other plant material, scavenging, invertebrates, and vertebrates	Percentage of each food category defines an important niche dimension and can reflect the niche breadth in terms of specialist and generalist. From the percentage food category, it possible to infer to which ecological processes each species is most likely to be linked (i.e., seed dispersal, pollination, removal of carcasses, controlling invertebrates, regulation of vertebrates	Wilman et al. (2014)
Foraging strata (three items)	Continuous	Indicates whether foraging stratum estimates are based on species level data: ground, understory, and canopy	Foraging strata relate to the location of resource acquisition	Wilman et al. (2014)
Body mass	Continuous	Based on the average of adult body mass (g)	Ecologists think about diet niche breadth in terms of prey size range, and the general pattern observed was that prey size tends to be directly proportional to the size of the predator, both within and between species	Wilman et al. (2014)
Dependence on forested habitat^a^	Categorical	Response trait to habitat loss and fragmentation (categories: high, medium, and low forest dependence)	Forest dependence reflects environmental tolerances, habitat, or ecological preferences of bird species and, consequently, relates to the location of resource acquisition (Violle et al., 2007). In addition, the trait attribute varies in response to changes in habitat loss and fragmentation	http://datazone.birdlife.org/home

a Dependence on forested habitat: we used habitat specialization as one of the traits to calculate overall species functional diversity. For habitat specialization groups, we used this trait a priori to define each group and not to calculate functional diversity.

### 2.5 Response variables

We used two matrices, containing the relative abundance of bird species sampled in each riparian forest, and the key functional traits (Table 1) of those species, to calculate three components of Functional Diversity—Functional Redundancy, Functional Evenness, and Functional Richness. We calculated all indices in R software (R Core Team 2014) using the “picante,” “FD,” and “ade4” packages. We used these three components to relate Functional Diversity with environmental predictors because they did not show strong auto‐correlations between them (Spearman correlation, *r* < 0.5), and so provide independent information on the distribution of species in functional trait space (Mason et al., 2005):

*Functional Redundancy (FRed)*: this index is described by de Bello, Lepš, Lavorel, and Moretti (2007) and is defined as the extent to which a community is “saturated” with species with similar traits. If Functional Redundancy is zero, all species are functionally different. By contrast, if redundancy is at a maximum (i.e., 1), all species are functionally identical. We calculated this index as the difference between the Simpson index of species diversity and the Rao quadratic entropy diversity index (i.e., generalization of Functional Diversity), following de Bello et al. (2007).
*Functional Evenness (FEve)*: measures the degree to which the biomass of a community is distributed in niche space. For instance, lower Functional Evenness indicates that some parts of niche space are under‐utilized, and thus, communities have lower ecosystem functions as results of less efficient resource use (Mason et al., 2005).
*Functional Richness (FRic)*: FRic values represent the niche space occupied by the species present in a community. This space is a convex hull volume defined by linked extreme values of species traits. From this, an algorithm calculates the volume inside the hull (Supporting Information Table S2). Thus, communities composed of species with similar functional traits have lower FRic values (Villéger et al., 2008).

We also calculated Functional Group Richness (FGR), which represents the number of functional groups per riparian forest based on a dendrogram of species traits. We used Gower distances (15 height) to cluster species in this dendrogram (Supporting Information Figure S2, Laliberté & Legendre, 2010). Although the choice of cut‐off point in the dendrogram depends on many analytical decisions and may be arbitrary, we reduced this arbitrariness by checking each functional group *a posteriori*. We observed that species with traits values distance lower 15 height are associated with similar ecosystem functions (Supporting Information Table S2).

### 2.6 Data analyses and modeling

To analyze the relationship of FD metrics with riparian habitat and vegetation variables, we tested the relationships of dependent (FRed, FEve, FRich), and predictive, variables (riparian forest width, disturbance index, NDVI mean, NDVI range, and NDVI Coefficient β) using simple linear models (lm R function) (R Core Team 2014), and selected the best predictor variable based on Akaike information criterion (AIC, see below) (Bolker, 2008). Then, we looked for nonlinearity in the relationships among Functional Diversity components and riparian vegetation characteristics by comparing linear, saturating (monomolecular), and null models using AIC values. Linear models show an increasing or decreasing constant trend in the relationships between dependent and independent variables, while monomolecular model has increasing trends with a saturating state, so indicating the maximum value of the dependent variable has been reached (parameter *a* of the curve).

To assess fit and performance, we compared models using the Akaike information criterion corrected for small samples (AICc). We considered as best models those with the lowest AICc values. We calculated ΔAICc to measure the difference between a candidate and best models, and we accepted as plausible models those with ΔAIC values equal to or <2. For each model, we also calculated Akaike weights *wi*, with a range from 0 to 1 (those having values closer to 1 being the most plausible models), which measures the strength of evidence for each model relative to the entire models set. AICs, ΔAICs, and Akaike weights were calculated by using AICctab function, of “bbmle” package (Bolker, 2017). Finally, we checked visually the fit and residuals distribution of each model selected. We did not find any departures from model assumptions (Supporting Information Figures S1 and S2) (Zuur, Ieno, Walker, Saveliev, & Smith, 2009).

After selecting the best predictor for each FD metric and identifying the relationship among them, we used regression tree analysis to estimate the breakpoint in the nonconstant relationships (De'ath & Fabricius, 2000; Müller & Bütler, 2010). Regression tree analyses allow to estimate thresholds in nonlinear relationship among two variables by iteratively splitting a data set in two groups with minimum sum of squares and with significantly different mean predictor values (Quinn & Keough, 2002). Finally, we compared the number of species within the most important diet guild in our samples (frugivorous, insectivores, and omnivores) above‐ and below‐estimated thresholds (categorical variable). This comparison was made using GLM models with Poisson error distribution, which is considered a robust tool for count data analysis (St‐Pierre, Shikon, & Schneider, 2018).

## 3 RESULTS

We sampled 76 bird species, divided into nine functional groups (Supporting Information Table S2). Species richness per forest varied from 6 to 22 species (mean = 10.7 ± 4.9 *SD*), and the number of functional groups in our samples varied from 3 to 9 groups (mean = 5.2 ± 1.5 *SD*). The number of species greatly varied among functional group (mean = 8.44 ± 7.73 *SD*); then, we opted to collapse functional groups into feeding guilds. The insectivorous group was the most numerous group in our study, reaching 40 species distributed in all sites. Frugivorous species were found in 21 sites in a total of 15 species. We also found 12 omnivorous species present in 19 sites. Species that consumes different food items (e.g., predominantly carnivores, granivores, and nectarivores) were not sufficiently sampled and were not included in the analyses (Supporting Information Table S2).

We observed that FRed was most strongly associated with riparian forest width (Table 2). All other variables we used to predict Functional Redundancy variation showed lower AIC model selection criterion support (Table 2). In turn, FEve was more associated with forest disturbance than either riparian forest width or the other three predictive variables we included in the model comparison (Table 2). Finally, FRic was related to both NDVI coefficient, NDVI mean, and disturbance level (Table 2), but the null model was also a plausible explanation for the data. Thus, the presence of the null model among the best models set indicated that variation in this response variable can be not related with any of the tested variables (Table 2).

**Table 2:** Model selection results for relationships of functional metrics (redundancy, evenness and richness) and riparian variables (riparian forest width and four disturbance indices)

Functional metric	Model effects	AICc	dAICc	*K*	*w*_i_
Functional redundancy	log(Riparian forest width)	−83.5	0	3	0.941
	NDVI range	−76.8	6.7	3	0.032
	NDVI mean	−74.2	9.3	3	0.009
	NULL	−73.9	9.6	2	0.008
	NDVI coef	−73.7	9.8	3	0.007
	Disturbance	−72.1	11.4	3	0.003
Functional evenness	Disturbance	−50.2	0	3	0.698
	log(Riparian forest width)	−46.6	3.5	3	0.119
	NDVI mean	−45.8	4.4	3	0.076
	NDVI coef	−44.9	5.3	3	0.049
	NULL	−44.4	5.8	2	0.039
	NDVI range	−43	7.2	3	0.019
Functional richness	NDVI coef	147	0	3	0.284
	NULL	147	0	2	0.283
	Disturbance	149	1.5	3	0.136
	NDVI mean	149	1.7	3	0.123
	log(Riparian forest width)	150	2.5	3	0.099
	NDVI range	150	2.6	3	0.076
Functional group richness	NULL	93.0	0.0	2	0.28
	NDVI coef	93.6	0.6	3	0.20
	Disturbance	93.9	0.9	3	0.17
	NDVI mean	94.3	1.3	3	0.14
	log(Riparian forest width)	94.8	1.8	3	0.11
	NDVI range	95.6	2.6	3	0.07

**Figure 2:**
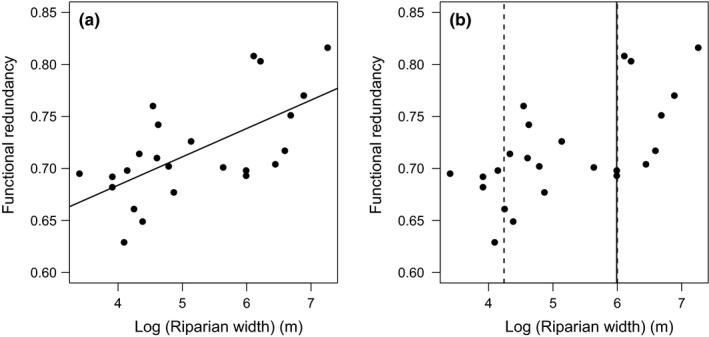
(a) Fit of the best model (Linear) to represent Functional Redundancy and Riparian forest width relationships. (b) Threshold estimate for the relationship between variables (solid lines) and 95% CI for that estimate (dashed lines). In both graphs, the black dots represent the observed data

**Figure 3:**
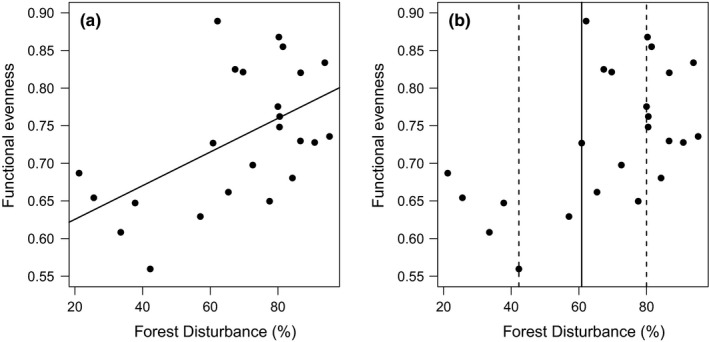
(a) Fit of the best model (Linear) for Functional Evenness and Riparian forest percentage disturbance. (b) Threshold estimate for the relationship between variables (solid lines) and 95% CI for that estimate (dashed lines). In both graphs, the black dots represent the observed data

After we selected the best predictive variables for FRed and FEve (riparian forest width, and forest disturbance, respectively), we found that a linear relationship was considered most plausible explanation for both variables (Table 3). Linear models showed lower AIC than saturating and null models for both Functional Diversity variables. Functional Redundancy had a positive linear relationship with riparian forest width, as we had initially expected. In turn, Functional Evenness also responds positively to disturbance, but the positive effect of disturbance on Functional Evenness was contrary to our initial expectations (Figures 2a and 3a).

**Table 3:** Model selection for type of relationships of functional redundancy and functional evenness, and the independent variables riparian forest width and forest disturbance, respectively. Columns names give the functional diversity metric, type of relationship, habitat variable, number of parameters (*K*), AICc and ∆AICc values and Akaike weights of each model

FD metric	Model	Variable	AICc	dAICc	*df*	*w*_i_
Redundancy	Linear	log(Riparian forest width)	−83.5	0.0	3	0.75
	Monomolecular	log(Riparian forest width)	−81.2	2.3	3	0.24
	Null	Constant	−73.9	9.6	2	<0.01
Evenness	Linear	Disturbance	−50.2	0.0	3	0.83
	Monomolecular	Disturbance	−46.4	3.8	3	0.12
	Null	Constant	−44.4	5.8	2	0.04

**Figure 4:**
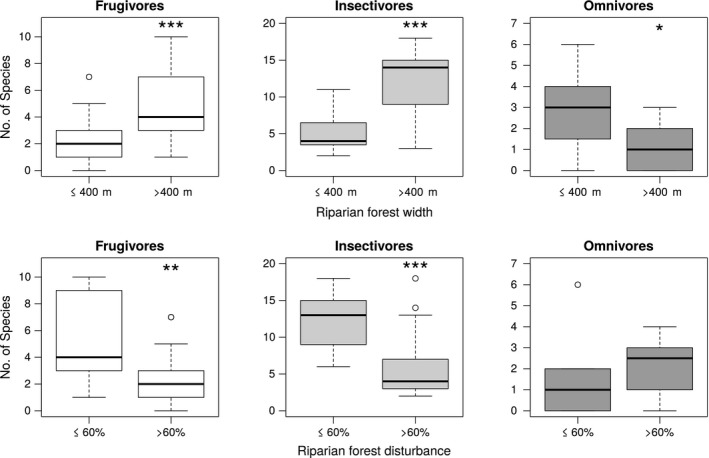
Boxplots for the number of species from the three most important feeding guilds found in riparian forests sampled in this study. Upper panels show number of species of Frugivores, Insectivores, and Omnivores below and above the 400‐m riparian forest width threshold. Bottom panels show the number of species of these three feeding guilds below and above the 60% riparian forest disturbance threshold. *significant difference at *p *<* *0.05; **significant difference at *p *<* *0.01; ***significant difference at *p* < 0.001

Regression tree analyses for these two relationships indicated that FRed was significantly different in riparian forests communities with widths below and above 400 m (estimated breakpoint = log(5.991)m (CI 95% = log(4.24)—log(5.99), Figures 2b and Supporting Information S3) and reached Fred‐predicted values of 0.695 and 0.767 below and above the threshold. On the other hand, FEve was significantly different below and above 60% disturbance (estimated breakpoint = 60.82% [CI 95% = 42.26–80.0], Figures 3b and Supporting Information S4) and reached predicted values of 0.644 and 0.769, respectively, in riparian habitats that had less or more than 60% in disturbance. Finally, we found that the number of frugivores and insectivores species was also greater above thresholds of riparian forest width and riparian forest disturbance (Figure 4) while omnivores did not show strong statistical differences in the number of species between communities located below and above both of the thresholds we estimated (Figure 4).

## 4 DISCUSSION

We have shown that Functional Redundancy and Functional Evenness in bird communities were affected by width and disturbance of riparian habitats, respectively, and those FD components showed a linear relationship with riparian forest changes. However, we identified thresholds along explanatory variables that were associated with abrupt changes in FD values and also in frugivores and insectivores species richness. Functional Redundancy and frugivores and insectivores richness declined with a reduction in riparian forest width, with the estimated threshold being around 400 m. Functional Evenness increased and frugivores and insectivores richness decreased when disturbance in riparian forests reached 60%. However, Functional Richness and number of omnivorous species were not affected in any way by any of the riparian changes we measured, with exception of a positive effect of riparian forest width reduction on the number of omnivorous richness. These results are consistent with those of other studies concerning habitat fragmentation effects on Functional Diversity (De Frutos, Navarro, Pueyo, & Alados, 2015; Liu et al., 2018; Magnago et al., 2014), but they also allow us to advance the topic by providing suggestions on how riparian habitats ecosystems responds to habitat alteration.

Decline in Functional Redundancy with riparian forest width reduction can be related to loss of functionally similar species, as the number of frugivores and insectivores species also shows a decline when riparian forests are narrow. This species loss could be due to the simplification of vegetation structure and the intense edge effects that occur in narrower riparian forests (Banks‐Leite, Ewers, & Metzger, 2010; Morrison, Lindell, Holl, & Zahawi, 2010). More simplified vegetation structures offer a lower availability and variety of resources, resulting in lower coexistence and lower number of species (Haugaasen & Peres, 2005; Rotenberry & Wiens, 1980). Our results suggest that these community changes may be stronger in riparian forests <400 m in width, where the impact of Functional Redundancy may compromise ecosystem resilience (Standish et al., 2014). In this sense, the linear relationship between Functional Redundancy and riparian forest width that we found indicates that bird communities in our study area had fewer redundant species and any loss of species would impact Functional Redundancy and ecosystem resilience (Schwartz et al., 2000). Otherwise, as other studies had shown, if the relationship between Functional Redundancy and riparian forest width represented a saturated state, then even the loss of several species would not promptly impact ecosystem function (Gerisch, Agostinelli, Henle, & Dziock, 2012; Naeem et al., 1998; Pillar et al., 2013). Therefore, to clearly understand the effect of habitat destruction and alteration on resilience, future studies need to explore the response diversity, and assay if (and how) functionally similar species respond differently to disturbance (Coelho, Raniero, Silva, & Hasui, 2016; Elmqvist et al., 2003).

On the other hand, that Functional Evenness increases with disturbance intensification indicates an increment in regularity in the distribution of species abundances in the functional space when disturbance is high (Mouchet, Villéger, Mason, & Mouillot, 2010; Mouillot et al., 2013; Villéger et al., 2008). Probably, this occurs because disturbance frequently modifies biotic interactions, destroying and altering habitat structure, and reducing resource availability (Aleixo, 1999; Mouchet et al., 2010; Sasaki, Lesbarreres, Watson, & Litzgus, 2015). All these alterations may change the relative importance of environmental filters, restricting the dominance of forest specialist species and favoring the colonization and/or increments in abundance of the habitat generalist species (Devictor, Julliard, & Jiguet, 2008; Ondine, Jean, & Romain, 2009; Pakeman, 2011). Indeed, we observed that omnivore species richness was greater in more disturbed sites and also in narrower riparian habitats. Thus, the addition of these species and trait states by disturbance can increase Functional Evenness (Biswas & Mallik, 2010; Mouchet et al., 2010), and we suggest that this change in community composition will happen when riparian habitats approach 60% disturbance.

The lack of effect of riparian forest changes (i.e., disturbance and width of riparian forest) on Functional Richness may occur because there is a high variability in forest dependence within our functional groups (Supporting Information Table S2). This variability could increase the capacity of the group to maintain its function in the face of riparian changes. In addition, such changes may be not strong enough to cause local extinction of those species with extreme traits values and, consequently, the volume of functional space remains constant (Mouillot et al., 2013).

### 4.1 Conservation and management implications

Is there a minimum riparian forests width or disturbance intensity that will have a minimum the impact on Functional Diversity? Our study bird communities did not tolerate reduction in riparian forest width or disturbance intensification without negative impact on Functional Diversity, and the effects are especially marked in widths below 400 m or when disturbance levels exceed 60%. These riparian changes compromise ecosystem resilience and function, exposing it to a high risk of normally provided ecological services loss and shift to an undesirable state to human welfare. However, although that answer has useful applications for land‐managers and policy‐makers, we would like to highlight some points. (a) These results should be viewed with caution, because we analyzed only bird communities and the relationships observed are dependent on considered traits. Thus, it is important to replicate this study to other taxonomic groups and with multiple ecological functions, before using any derived thresholds as regulatory limits for decision‐making processes. (b) It is quite unrealistic to think only in terms of the width of riparian forest as a means of protecting Functional Diversity. To properly conserve Functional Diversity, it is important go beyond considering the disturbance intensity of riparian forests. (c) Even if changes to riparian forests have no impact on some functional components, such as Functional Richness, there is no guarantee that all other components will have the same neutral response. Thus, it is too dangerous to use regulatory limits for decision‐making processes in relation to riparian changes, based on single component of Functional Diversity.

Despite this, previous studies have already established a minimum width for riparian forests for conservation and restoration activities, but such studies have been focused on traditional measures of species diversity that include only information about the presence or abundance of species (200 m in Lees & Peres, 2008; 100 m in Metzger, 2010a,b; 120 m wide in Tubelis et al., 2004). We consider the distribution of ecological traits in the community to gain a deeper understanding of the role species in ecosystem processes and how to maintain effective ecosystem resilience and the services that the biological diversity provides. Then, our results indicated that Functional Diversity possibly is more promptly affected than the more standard measures of taxonomic diversity.

Overall, it is important to emphasize that several countries have specific sets of laws to protect riparian forest (Lee, Smyth, & Boutin, 2004). In Brazil, environmental legislation for private land (New Forest Code (Law 12,651/2012: available at: http://saema.com.br/files/Novo%20Codigo%20Florestal.pdf) mandates that all rural landholdings must protect those riparian forest bordering streams. The width of the riparian forest varies according to the width of the streams. For streams <10‐m wide, the Forest Code designates a protected area that is 15‐m wide on each side of the stream. This law has been criticized by the scientific community, because such a forest width is too small to protect most terrestrial forest specialist species (Bueno et al., 2012; Hawes, Barlow, Gardner, & Peres, 2008; Marczak et al., 2010; Metzger, 2010b; Metzger et al., 2010).

Therefore, this law unfortunately does not safeguard the minimal extent of riparian forests that would be required to protect the Functional Diversity and, consequently, adherence to the news laws may severely compromise ecosystem resilience and function. Considering the economic values of ecosystem services, it is important to encourage landowners not only to maintain or restore riparian forests, but also to reduce disturbance events (e.g., fire, grazing, livestock drinking sites, selective logging, and exotic plant invasion (e.g., *Brachiaria*). These events often destroy or remove biomass and hinder or halt forest regeneration, which is essential to restore habitat structure (Lees & Peres, 2008). Encouraging better management practices can be achieved by providing, for example, payments for ecosystem services provided (Banks‐Leite et al., 2014). Some such incentives have already been implemented in some localities in Atlantic Forest, Brazil, beginning in 2005, and growing rapidly in scope and popularity since then (Taffarello, Calijuri, Viani, Marengo, & Mendiondo, 2017). However, these incentives are still focusing on watershed protection and efforts are needed to expand into other ecosystem functions and services, such as seed dispersal and pest control.

## CONFLICT OF INTEREST

None declared

## AUTHOR CONTRIBUTION

LAM and EH conceived and designed the study. LAM, AVA, BFCBA, DLS, ELA, RMT, and RMG conducted field work and previous analyses. EH and RCR conduced current analyses. LAM, EH, and RCR wrote the manuscript and provide editorial requirements.

## DATA ACCESSIBILITY

Riparian forest and bird data are available at https://doi.org/10.5061/dryad.g7253h2.
