# Supplementary figures and images for: Functional Redundancy in bird community decreases with riparian forest width reduction
Source: Ecol Evol. 2018 Oct 11;8(21):10395–408. doi: 10.1002/ece3.4448 (PMC6238144; doi:10.1002/ece3.4448)

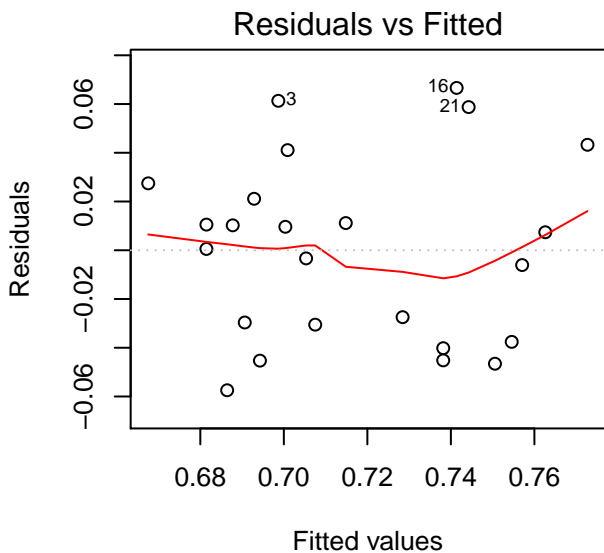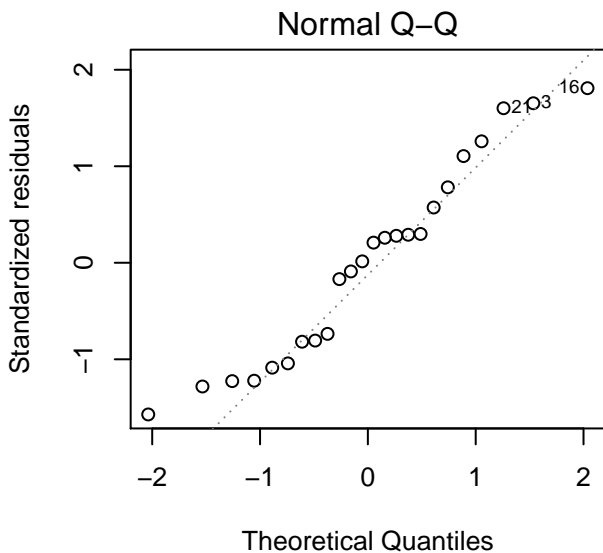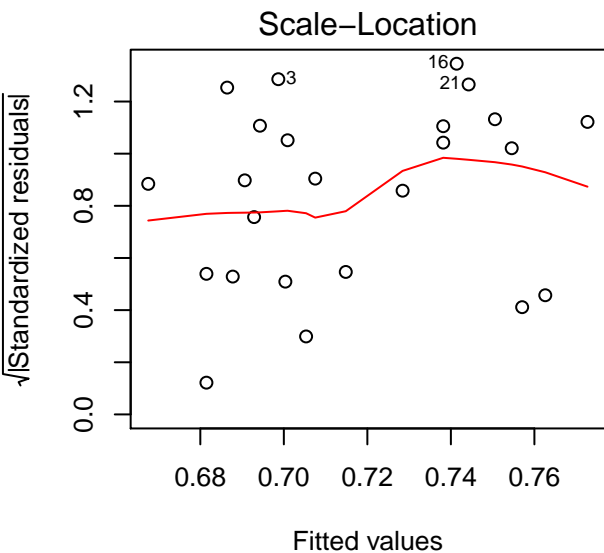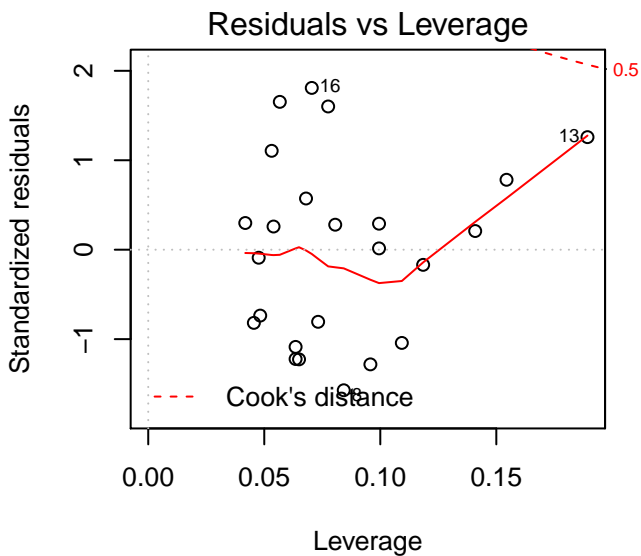

Supplement: Supplementary file 1 [file ECE3-8-10395-s001.pdf]

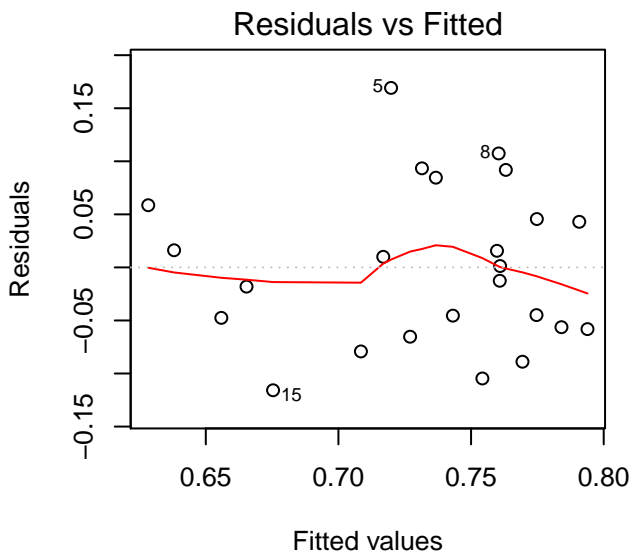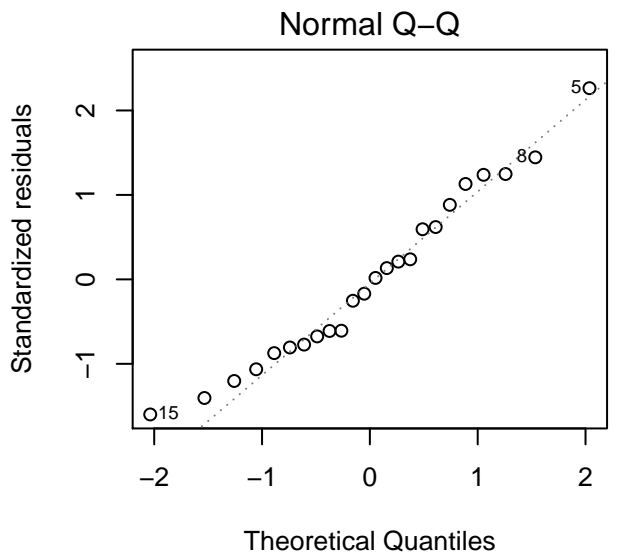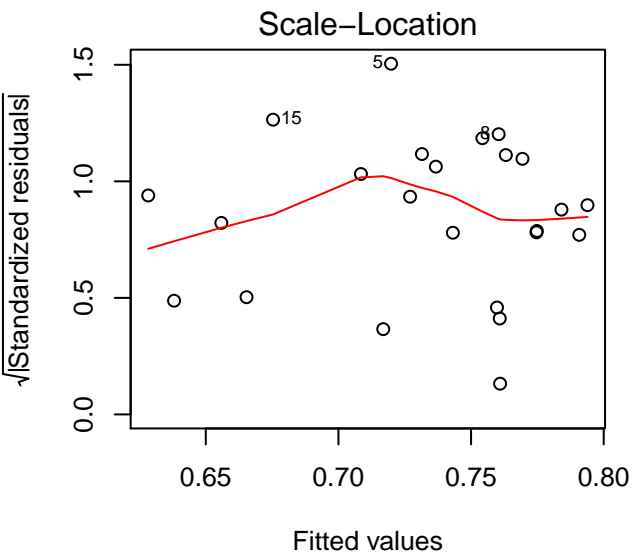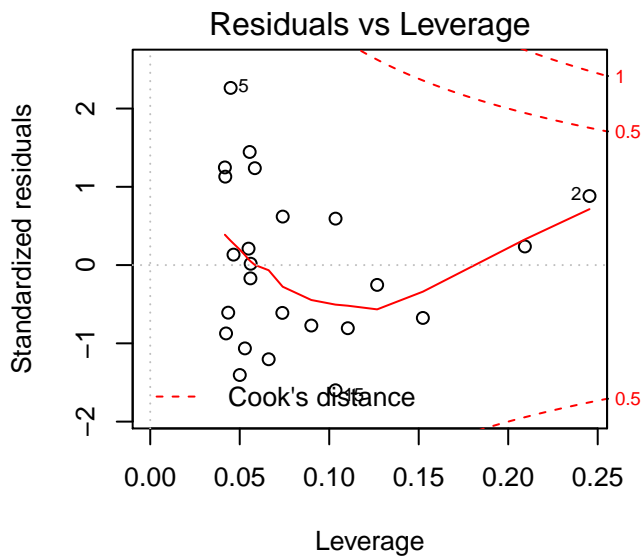

Supplement: Supplementary file 2 [file ECE3-8-10395-s002.pdf]
